# Human embryonic stem cell classification: random network with autoencoded feature extractor

**DOI:** 10.1117/1.JBO.26.5.052913

**Published:** 2021-04-29

**Authors:** Benjamin X. Guan, Bir Bhanu, Rajkumar Theagarajan, Hengyue Liu, Prue Talbot, Nikki Weng

**Affiliations:** a University of California–Riverside, Center for Research in Intelligent Systems, Riverside, California, United States; b University of California–Riverside, Stem Cell Center, Riverside, California, United States

**Keywords:** bioinformatics, cell classification, human embryonic stem cell, phase contrast videos

## Abstract

**Significance**: Automated understanding of human embryonic stem cell (hESC) videos is essential for the quantified analysis and classification of various states of hESCs and their health for diverse applications in regenerative medicine.

**Aim**: This paper aims to develop an ensemble method and bagging of deep learning classifiers as a model for hESC classification on a video dataset collected using a phase contrast microscope.

**Approach**: The paper describes a deep learning-based random network (RandNet) with an autoencoded feature extractor for the classification of hESCs into six different classes, namely, (1) cell clusters, (2) debris, (3) unattached cells, (4) attached cells, (5) dynamically blebbing cells, and (6) apoptotically blebbing cells. The approach uses unlabeled data to pre-train the autoencoder network and fine-tunes it using the available annotated data.

**Results**: The proposed approach achieves a classification accuracy of 97.23±0.94% and outperforms the state-of-the-art methods. Additionally, the approach has a very low training cost compared with the other deep-learning-based approaches, and it can be used as a tool for annotating new videos, saving enormous hours of manual labor.

**Conclusions**: RandNet is an efficient and effective method that uses a combination of subnetworks trained using both labeled and unlabeled data to classify hESC images.

## 1 Introduction

Human embryonic stem cells (hESCs) are derived from the inner cell mass of developing blastocysts and possess two important properties: (1) self-renewal and (2) pluripotency.^1^^–^^3^ Self-renewal is the ability to go through unlimited cycles of cell division, and pluripotency is the capability to differentiate into any cell type in the human body. hESCs are an important resource for regenerative medicine, basic research on human prenatal development, and toxicological testing of drugs and environmental chemicals. Under their state of pluripotency, they can also be maintained indefinitely.^4^^,^^5^ hESC classification is an important task for toxicity studies. Through classification of hESCs in time-lapsed videos, biologists can analyze apoptotic behaviors in both cell clusters and individual cells under certain test chemicals. Therefore, understanding the behavior of hESCs is fundamental for medicinal and toxicological research.^5^^–^^8^

**Fig. 1:**
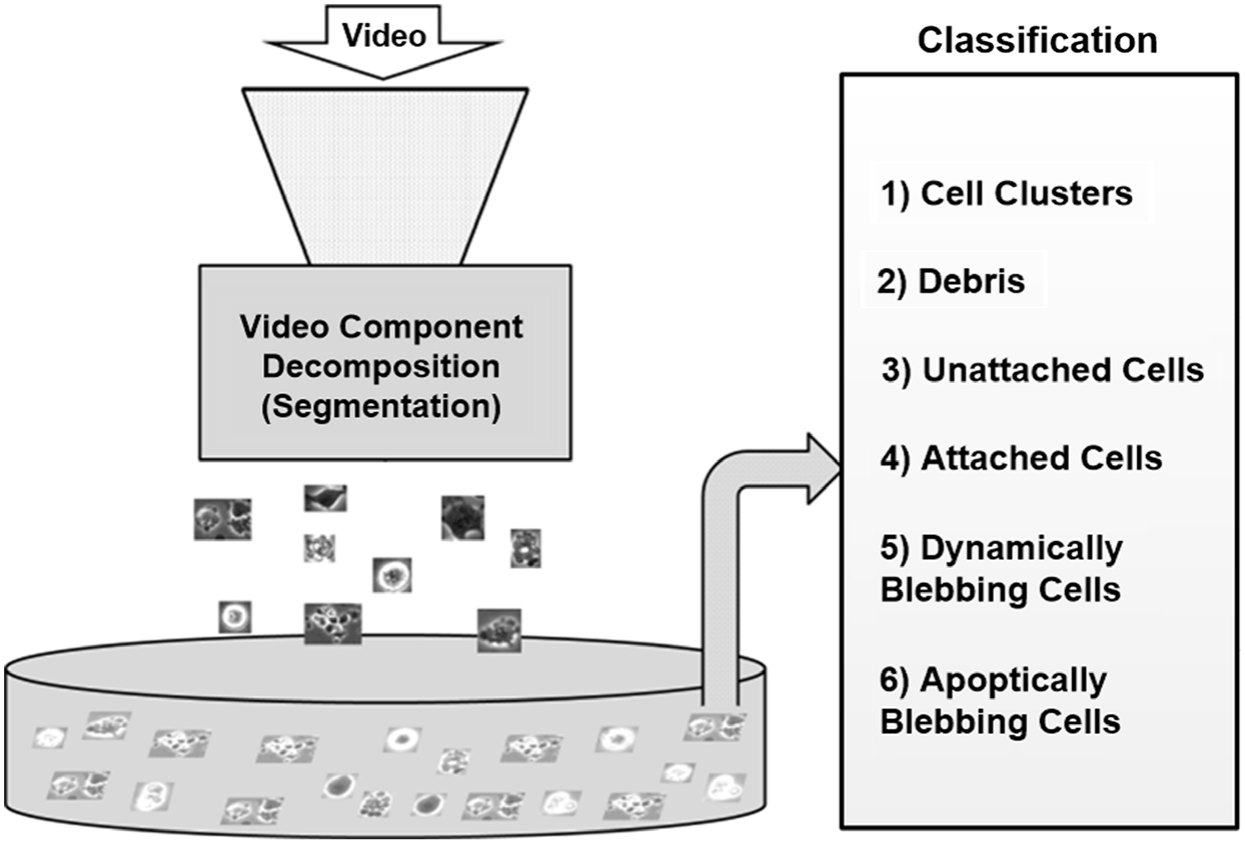
System overview.

The classification of hESCs in video is essential for quantifiable analysis of hESC processes and behavior.^9^ However, manual analysis of stem cells is laborious, tedious, and often inaccurate due to three main human limitations. First, the accuracy of a human performing classification is inversely proportional to long working hours. Second, uncertainty in classification occurs due to a wide variety of objects that appear in a class. Third, the amount of time put into working on datasets can lead to confusion in classifying hESCs into the right classes. Figure 1 shows a modularized system overview for an automated segmentation and classification process. In this paper, we focus essentially on the classification of the detected components from hESC videos; the detected components are the six general classes shown in Fig. 1. Guan et al.^3^ provide details of a method for the fast detection and segmentation of individual video components.

**Fig. 2:**
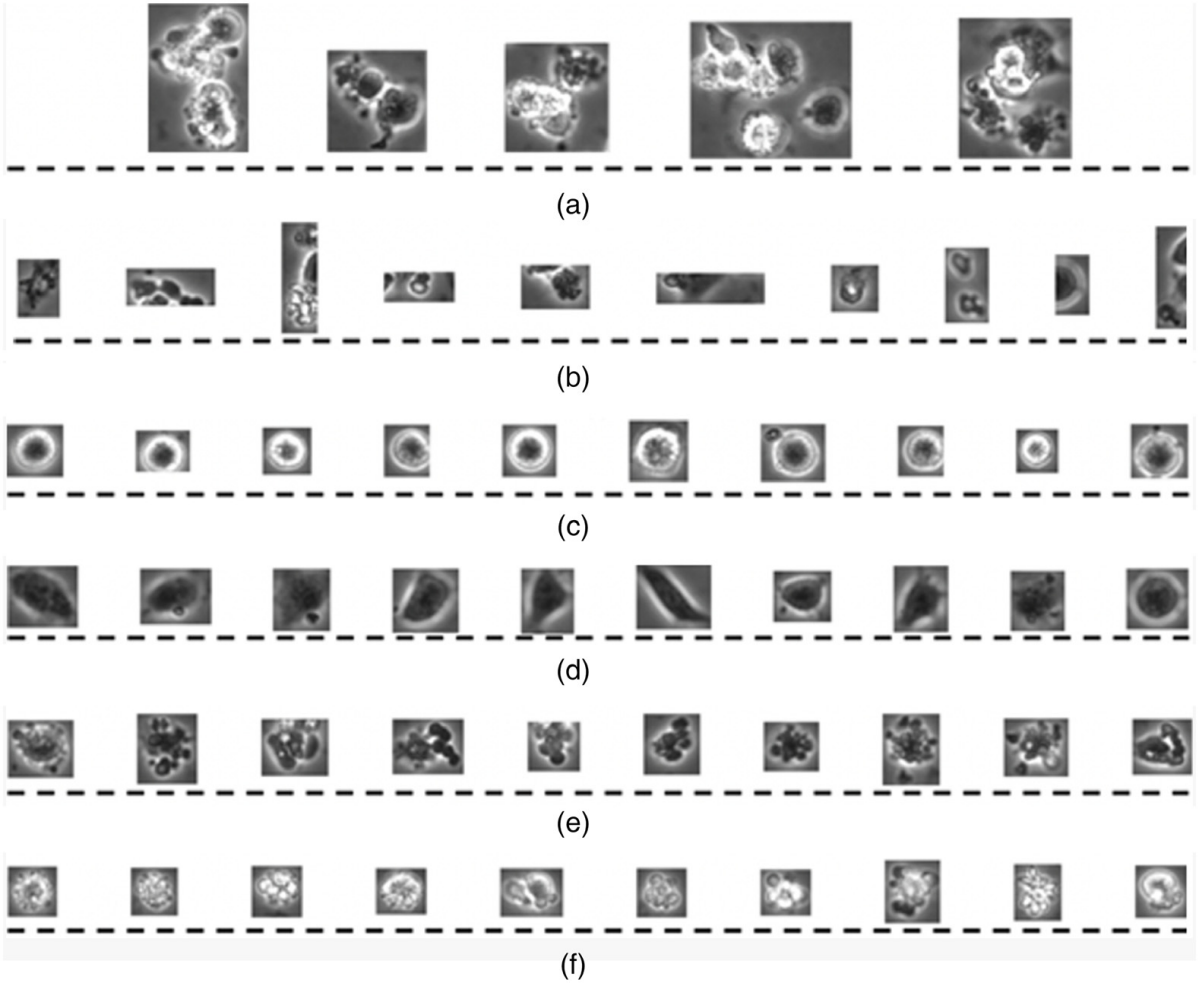
Six classes of hESCs from phase contrast images detected using the approach proposed by Guan et al:^3^ (a) cell clusters; (b) debris; (c) unattached cells; (d) attached cells; (e) dynamically blebbing cells; (f) apoptotically blebbing cells. It is to be noted that the cells are going through multiple states during the data collection (at every few minutes), which could last for 48 to 100 h.

Because phase contrast imaging is a non-invasive microscopy technique, it is widely used to study the behavior of live hESCs in video.^10^ In this study, the hESC videos were taken with a BioStation IM.^11^ The Biostation has an incubator with time-lapsed video capability. Each video captures an assay. The BioStation IM enables video capture of living cells under a stable and optimal environment. More details about BioStation IM and the images can be found in Talbot et al.^7^ The hESC videos consist of frames of phase contrast images. Each frame can contain any of the following six general components: (1) cell clusters, (2) debris, (3) unattached cells, (4) attached cells, (5) dynamically blebbing cells, and (6) apoptotically blebbing cells. Among these unattached, attached, dynamically blebbing, and apoptotically blebbing cells are the four classes that are of significant interest in experimental work. These four classes are regarded as the four intrinsic cell types in a video. Figure 2 shows examples of the six classes. Conceptually, the six classes of hESCs can be distinguished with three fundamental human perceptual capabilities for identification and classification of objects: (1) shape, (2) intensity, and (3) texture. Each class can be uniquely identified by one or a combination of the aforementioned human perceptions. For instance, the apoptotically blebbing cells in Fig. 2(f) are similar in intensity, shape, and texture among themselves. hESCs in Figs. 2(e) and 2(f) are dissimilar in intensity, but they are similar in shape and texture. The debris in Fig. 2(b) has similar intensity values as various classes shown in Fig. 2. Traditionally, a feature vector can be derived with the aforementioned human perceptions. However, with the advent of deep learning techniques, we can develop classification models with the given abundance of labeled data. Therefore, the need to generate a feature vector manually for a classification system is only suitable when data are quite limited.

With the consideration that we often see an abundance of unlabeled data rather than labeled data, we propose a random network (RandNet) with an autoencoded feature extractor. The proposed method focuses on building random subnetworks with the feature extractor derived from unlabeled data. Moreover, the proposed method incorporated ensemble methodology in the network to reduce overfitting.

### 1.1 Related Work

**Fig. 3:**
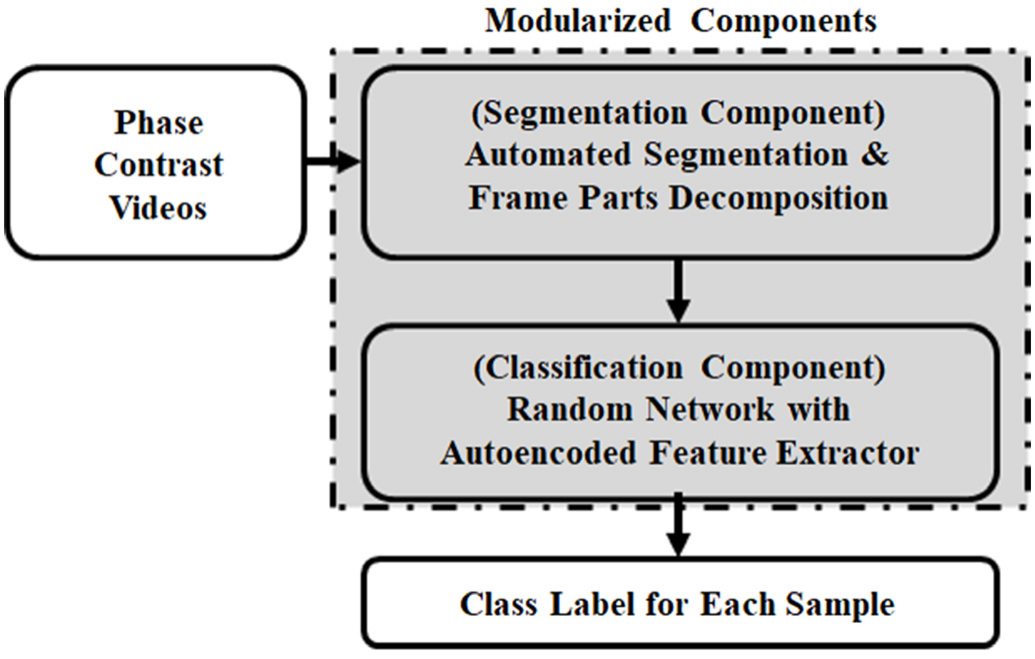
Automated segmentation and classification system overview.

To develop a practical system with high classification accuracy, modularization structure is often preferred over a deep learning approach that simultaneously performs detection and segmentation because modularized components allow for flexibility and adaptability as shown in Fig. 3 and Refs. 12–14. We consider segmentation and classification to be two separate modularized components or subsystems. Additionally, direct classification from the input videos is extremely challenging because these are dynamic images evolving over time.

In this paper, we focus on the classification component. There has been very limited work on building an automated classification system for stem cells in video with both labeled and unlabeled datasets.^8^ Niioka et al.^15^ used convolutional neural network (CNN) to study cellular differentiation from myoblasts to myotubes. Their classification model was built upon the concept that cellular morphology changes during differentiation, and this feature was easily captured in stained fluorescent images. In addition, Xie et al.^16^ worked on fluorescent images with CNN for cell counting. Although they have a successful experiment, their classification problem was simple since their images contained only circular dots. Chang et al.^17^ also used CNN for human induced pluripotent stem cell regions classification.^17^ Their study focused on classifying cell cluster patterns. The dataset used in the works by Niioka et al.,^15^ Xie et al.,^16^ and Chang et al.^17^ came from experiments that use staining techniques; staining is a very intrusive technique to be used on cells for contrast enhancement. However, our hESC experiments were done without staining.

Similar work on stem cell classification with phase contrast images was proposed by Theagarajan et al.^18^^,^^19^ They suggested using a generative method to train the network and classify real data. However, they did not consider realistic unlabeled data, which can be efficiently generated for training; typical generative methods have huge computational cost for synthetic dataset generation as well as training with a large set of synthetic data. Therefore, this paper proposes using the unlabeled data (without the use of generative methods) for model training and fine-tuning the model with labeled data.

### 1.2 Contributions of this Paper

In this paper, we focus on the classification component. From Fig. 2, we can infer that there are four major challenges in hESC classification. First, when attached cells spread thin in the substrate, the cells are fused with the background. Second, dynamically blebbing cells and apoptotically blebbing cells are similar in intensity. Third, when a large attached cell goes through the apoptotic process, it appears as a cell cluster of apoptotically blebbing cells. Fourth, image data are obtained under both 10× and 20× objectives, which adds challenges in discerning individual blebbing cells from cell clusters. In light of the state of the art, the contributions of this paper are as follows.

• We introduce the concept of creating a modularized system to automatically segment and classify hESCs in video. This reduces the complexity of the problem since it is extremely challenging to classify hESCs directly from the video in a single step.
• We introduce the concept of building feature extractor with unlabeled data and unsupervised learning. Hence, we do not require huge amounts of labeled data as is required in deep learning based approaches.
• We incorporate ensemble methodology into the proposed RandNet to handle the diversity of data generated during the experiments that last at least 48 to 100 h. We are not aware of any such work in biological image analysis.
• We provide experimental results and comprehensive comparison with state-of-the-art techniques.

Section 2 presents the materials and methods in detail. Section 3 provides experimental results, and Sec. 4 provides a discussion on the proposed and compared methods. Finally, Sec. 5 presents the conclusions of the paper.

## 2 Materials and Methods

### 2.1 Materials

All time lapse videos were obtained with the phase contrast microscope in BioStation IM.^7^^,^^11^ The videos were acquired using either a 10× or 20× objective with 600×800  pixel resolution. A total of 27,603 unlabeled gray scale images and 3559 labeled gray scale images were obtained from six 10× videos and eight 20× videos. Both unlabeled and labeled images were obtained automatically by the method described in Guan et al.^3^^,^^20^^,^^21^ The labeled dataset had the following number of gray scale images for each class: (1) 636 cell cluster images, (2) 773 debris images, (3) 519 unattached cell images, (4) 704 attached cell images, (5) 413 dynamically blebbing cell images, and (6) 514 apoptotically blebbing cell images. The ground-truth for the datasets were generated manually by stem cell experts. We used 75% of the dataset for training and the remaining 25% of the dataset for out-of-sample testing for each class. To generalize the classifier, five-fold cross validation was done during model learning. Model learning is performed with training data only.

### 2.2 Methods

In this section, we first present the motivation for our proposed approach. This is followed by a method for automated cell region detection, which is the segmentation component. We then describe RandNe and elaborate on the autoencoded feature extractor as well as the pre-trained subnetworks for the classification component. The classification component is part of the modularized system as shown in Fig. 3. A pseudocode for building the RandNet model is also provided.

#### 2.2.1 Motivation of the approach

Domain knowledge often comes from human perception, which is the most complex yet efficient cognitive system. Through hypothetical assumeption and visual inspection, we can sometimes identify useful features of hESCs for classification. However, domain knowledge is limited by the amount of information the brain can absorb. With tens of thousands of unlabeled and labeled data, experts can have hard times in either conceptualizing or generalizing the hidden information contained in the data. Deep learning techniques can help to understand the vast amount of data and solve the difficulty in creating automated algorithms for repetitious tasks performed by humans. Consider the task of studying apoptotic processes of cells with test chemicals in a toxicity experiment. Observing the dynamic changes in the texture and shape of apoptotic processes of a cell requires a significant amount of manual labor for annotating individual video frames. Currently, biologists spend hours of manual labor in annotating these images, which is a very tedious and menial task. Our deep learning based approach can learn to automatically segment these frames from the vast amount of data available in an unsupervised manner, thus significantly reducing the amount of time biologists spend annotating images, which improves their efficiency. The proposed approach uses an unsupervised technique to build the foundation of the encoder network. The proposed method also uses of both the unlabeled and labeled data to build a reliable classification system.

#### 2.2.2 Segmentation component

**Fig. 4:**
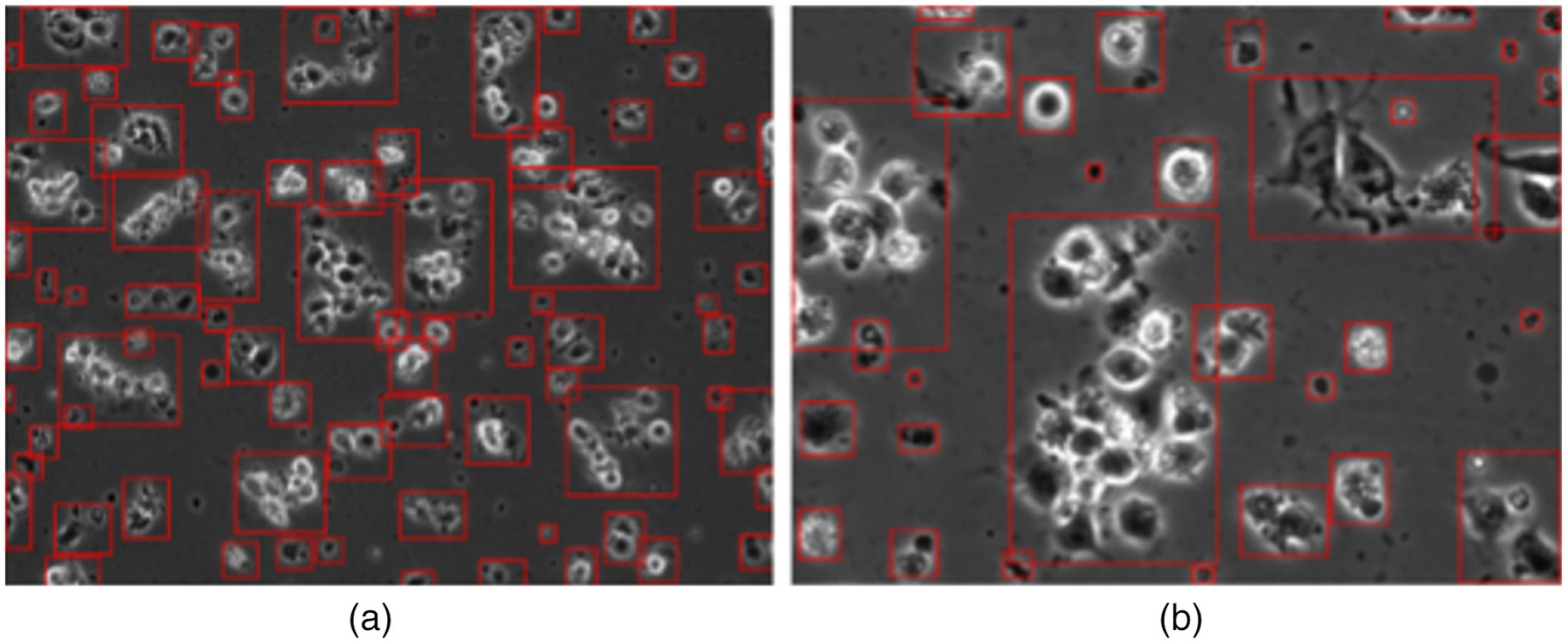
Detected components on each frame:^3^ (a) image under 10× objective and (b) image under 20× objective.

Guan et al.^3^ proposed a model based method for automatically segmenting hESCs. This automated cell region detection is an essential algorithm in developing automated frame component decomposition in hESC phase contrast videos. They considered the foreground and background intensity distribution to be a mixture of two Gaussians. The objective of their algorithm is to find an optimal threshold that optimizes a criterion derived from the intensity distribution of foreground and background. The optimal segmentation is achieved at the highest criterion value. Since the segmentation method yields a binary image for each frame, we were able to extract a pool of individual components from each frame. Figure 4 shows the detected components of frames under 10× and 20× objectives. These detected components are then ready to be classified into one of the six aforementioned classes.

#### 2.2.3 Classification system overview

**Fig. 5:**
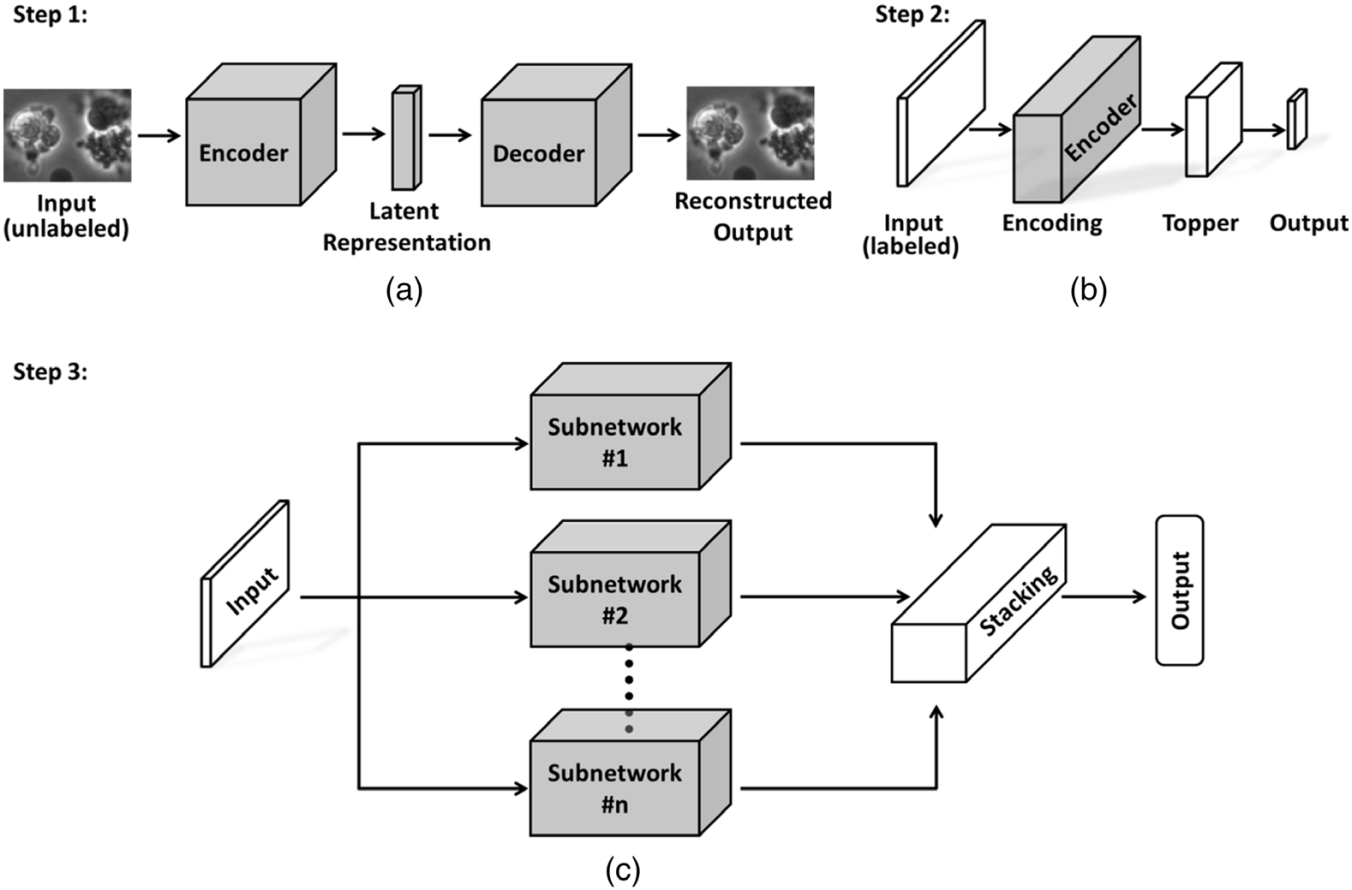
(a) Autoencoder network; (b) pre-trained subnetwork; (c) RandNet with autoencoded feature extractor.

The proposed classification system is built with both labeled and unlabeled data, and it consists of many random pre-trained subnetworks. The proposed method utilizes unlabeled data to build the encoder component in the pre-trained subnetworks and labeled data to fine-tune the RandNet. The RandNet structure also incorporates ensemble methodology to constrain overfitting. Figure 5 shows a graphical depiction of how RandNet is built with pre-trained subnetworks and the ensemble concept.

#### 2.2.4 Random network

**Fig. 6:**
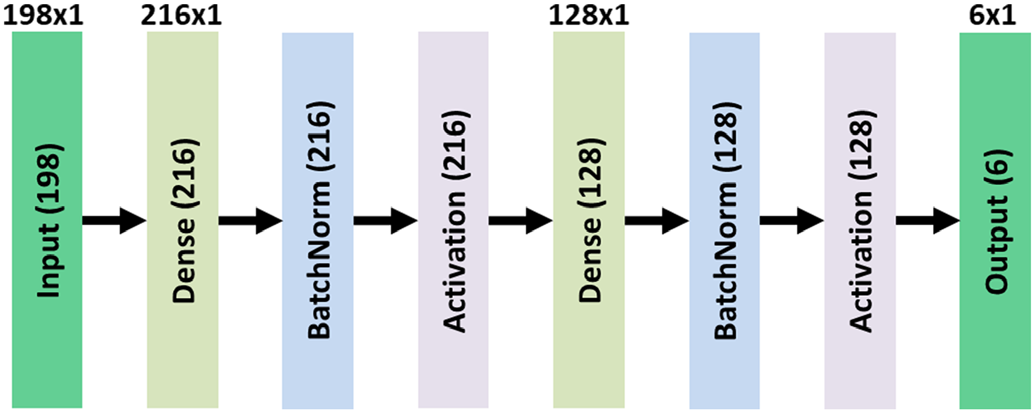
Stacking network. (Note: Dimensions without parentheses are kernel dimensions of the current box, and dimensions with brackets are output dimensions of the current box.)

RandNet utilizes the concept of bagging in deep learning by creating subnetworks. Bagging or bootstrap aggregation is a machine learning concept used to reduce variance and avoid overfitting.^22^^–^^25^ RandNet, developed in this paper, is a method that contains many subnetworks that have a common pre-trained model and are fine-tuned with random samples. RandNet uses all of the results from each subnetwork and passes it to a stacking network in which the final decision is made. The detail of the stacking network is shown in Fig. 6. The stacking network is designed to be simple and has only two main dense layers.

#### 2.2.5 Autoencoded feature extractor

**Fig. 7:**
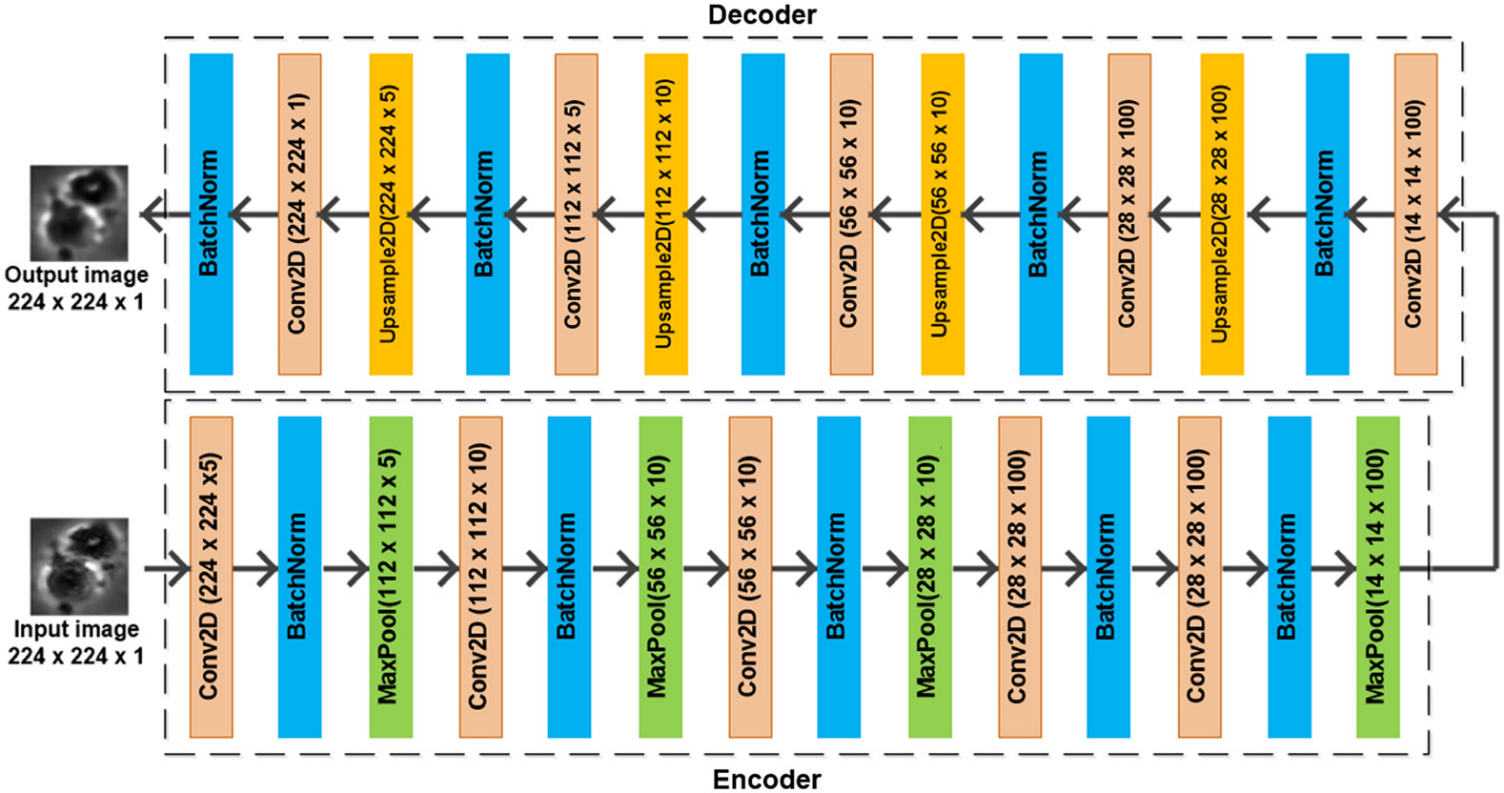
Architecture of the autoencoder network. (Note: Dimensions without parentheses are kernel dimensions of the current box, and dimensions with brackets are output dimensions of the current box.)

The autoencoder network is an efficient unsupervised learning method that learns the representation of a set of data. The autoencoder network contains two major components: encoder and decoder.^26^^–^^28^ In this paper, we used a structure similar to AlexNet as the basis of an encoder, and then we designed a decoder network from it. Although the VGG architecture^29^ slightly outperforms AlexNet^30^ as shown in Sec. 3.3, this difference is not significant, and since the AlexNet architecture requires reduced computational resources, we chose it for its simple implementation. As shown in Fig. 5(a), the encoder generates a set of latent representations for the unlabeled data. The details of both encoder and decoder structures are shown in Fig. 7. The autoencoder network used the Adadelta optimizer^31^ and the pixel-wise binary cross-entropy loss function. Since the final layer in the autoencoder network was chosen to be a sigmoid activation layer, pixel-wise binary cross entropy is an applicable loss measure. The loss function equation is given as follows:

$$Loss_{AE}=-\overset{N_{S}}{\underset{i=1}{\sum }}\overset{N_{R}}{\underset{r=1}{\sum }}\overset{N_{C}}{\underset{c=1}{\sum }}I^{\left(i\right)}(r,c)\log(K^{\left(i\right)}(r,c))+(1-I^{\left(i\right)}(r,c))\log(1-K^{\left(i\right)}(r,c)),$$ (1)

where $Loss_{AE}$ is the total pixel-wise loss in the autoencoder network, $N_{S}$ is the total number of sample images in a batch, and $N_{R}$ and $N_{C}$ are the total number of rows and columns, respectively. $I^{\left(i\right)}(r,c)$ and $K^{\left(i\right)}(r,c)$ are the ground-truth and predicted label values, respectively, in the r’th row and c’th column for the i’th sample. Both $I^{\left(i\right)}(r,c)$ and $K^{\left(i\right)}(r,c)\in [0,1]$.

#### 2.2.6 Pre-trained subnetwork

**Fig. 8:**
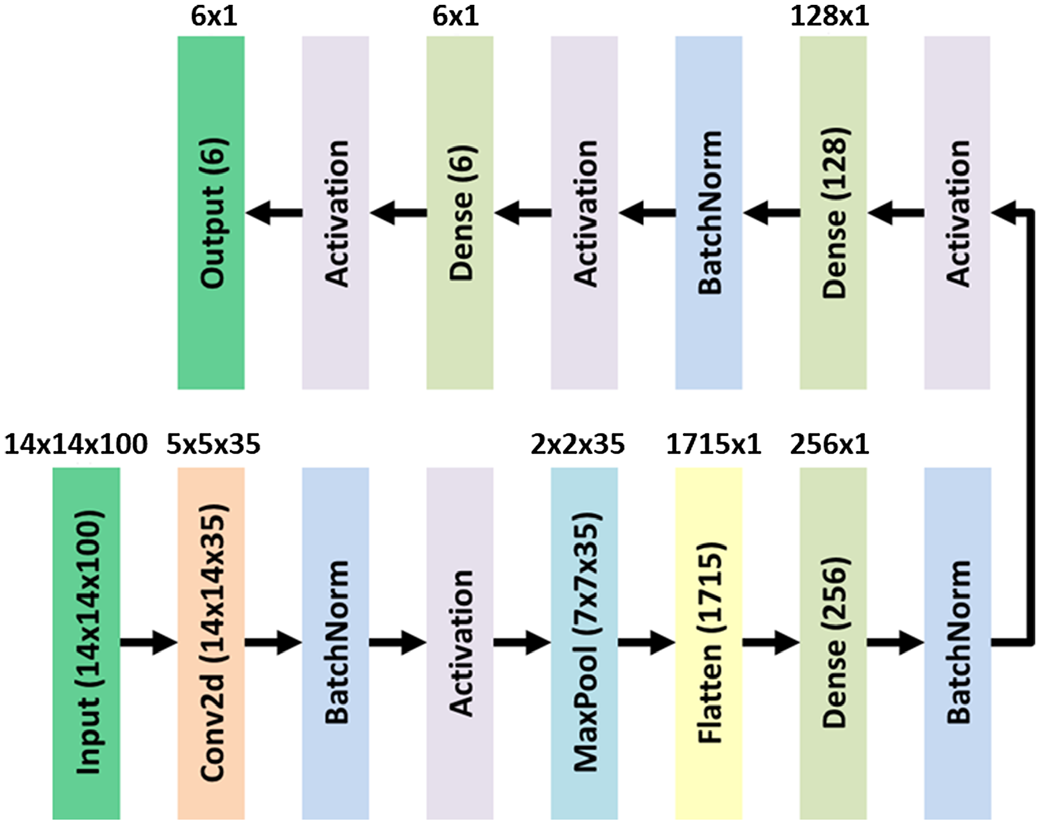
Topper structure. (Note: Dimensions without brackets are kernel dimensions of the current box, and dimensions with parentheses are output dimensions of the current box.)

The subnetwork used the encoder structure derived from the autoencoder network [in Step 2, Fig. 5(b)] as the basis for building a subclassifier. Each pre-trained subnetwork is fine-tuned with random samples and has a topper structure. The layers of the topper structure are shown in Fig. 8.

Since the encoder structure was unfrozen in each subnetwork, the fine-tuning with random samples affects the weights in the encoder structure. Therefore, we were able to emulate bagging for the proposed method. For this subnetwork, we use categorical cross entropy as our loss function, which is given as

$$Loss_{\mathrm{CCE}}=-\overset{N_{S}}{\underset{i=1}{\sum }}\overset{M}{\underset{j=1}{\sum }}y_{\left(i,j\right)}\;\log(p_{\left(i,j\right)}),$$ (2)

where $Loss_{\mathrm{CCE}}$ is the total categorical cross entropy in the pre-trained subnetwork. $N_{S}$ and M are the total number of samples images and classes in a batch, respectively. $y_{\left(i,j\right)}$ and $p_{\left(i,j\right)}$ are the ground-truth and predicted values, respectively, for i’th sample and j’th class, where, $y_{\left(i,j\right)}$ and $p_{\left(i,j\right)}\in \{0,1\}$. Table 1 shows the pseudocode for building the classifier model.

**Table 1:** Pseudocode for building the classifier model.

Algorithm 1: Random Network with Autoencoded Feature Extractor
**Input**: $D_{\text{unlabeled}}$ is a set of unlabeled data
$D_{\text{labeled}}$ is a set of labeled data
n is the total number of subnetworks
**Output**: Final learned model
1. **Procedure** RandNet($D_{\text{unlabeled}}$, $D_{\text{labeled}}$, n)
2. Normalize $D_{\text{unlabeled}}$ and $D_{\text{labeled}}$ by dividing by 255
3. Train Autoencoder network with unlabeled data [Step 1, Fig. 5(a)]
4. Extract encoder structures from autoencoder network for subnetwork training [Step 3, Fig. 5(b)]
5. Create n subset of labeled data with stratified bootstrap.^32^ These subsets are used to obtain n subnetworks
6. Fine-tune n subnetworks with the above datasets
7. Connect the output from n subnetworks with stacking technique [Step 3, Fig. 5(c)]^33^
8. Train the final network with all of the training data
9. Obtain the final model
10. **End procedure**

## 3 Results

### 3.1 Parameters and Optimization

**Fig. 9:**
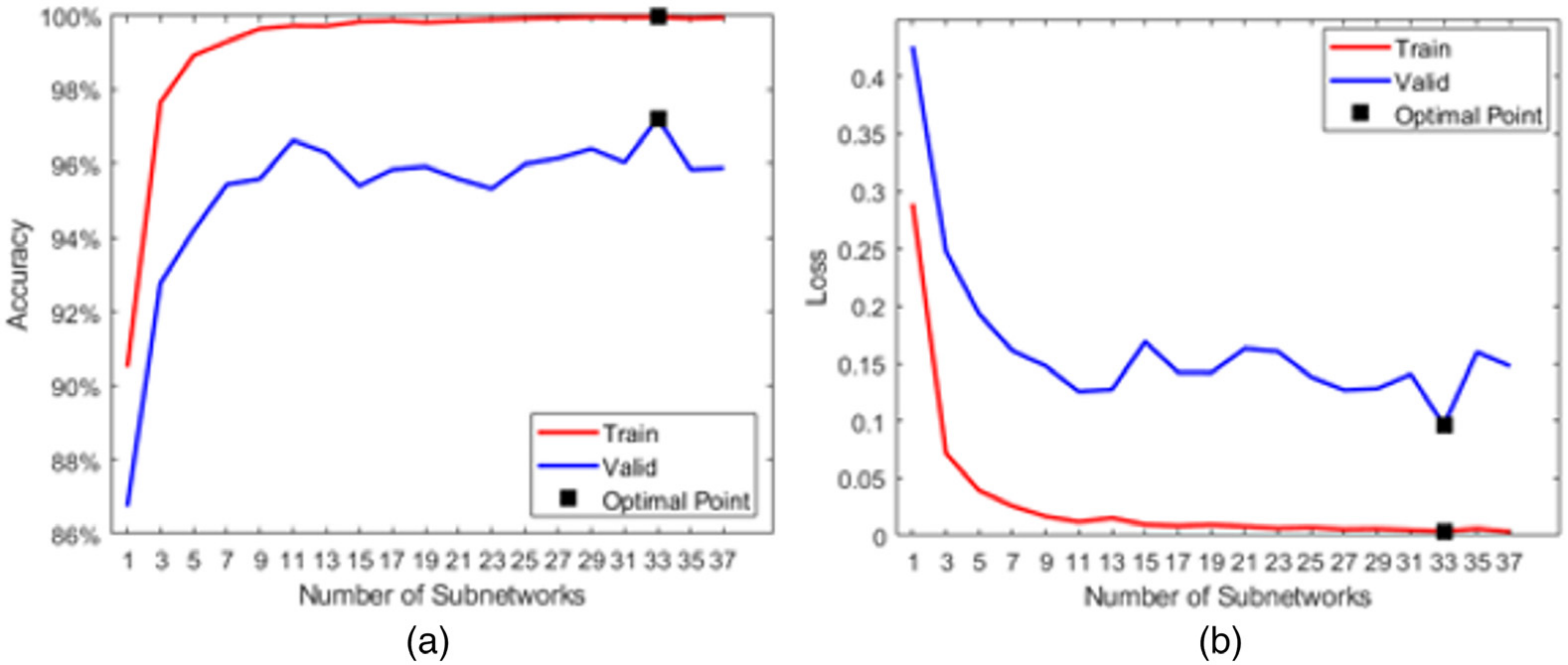
Five-fold cross-validation results. (a) Mean accuracy vs. number of subnetworks curve; (b) mean loss vs. number of subnetworks curve.

In our approach, all cropped images after the detection module were resized to 224×224 with bicubic interpolation, and the image intensities were normalized by dividing them by 255. No additional data augmentation was performed. For the autoencoder network, each subnetwork was trained independently, and the latent representation of the subnetwork was used to train the topper network. There are two fixed parameters for each subnetwork: epochs and batch size, which are set to be 10 and 128, respectively. The default Adadelta optimizer is used for the autoencoder network.^31^ For RandNet, there are five parameters: epochs, batch size, number of subnetworks, learning rate, and decay rate. We used 25 epochs with early stopping, a batch size of 50, and a total of 33 subnetworks. We also used a default Adam optimizer^34^ with the learning rate of 0.001. All parameters are fixed except the number of subnetworks, which has a search range from 1 to 37 with a step size of 2. Figure 9 shows that, when the number of subnetworks equals 33, it has the highest average validation accuracy as well as the lowest average validation loss. It should also be noted that the processing speed for our approach using all 33 subnetworks during inference is 6.25 frames per second (FPS) compared with 4.16 FPS using the approach proposed by Theagarajan et al.^19^

**Fig. 10:**
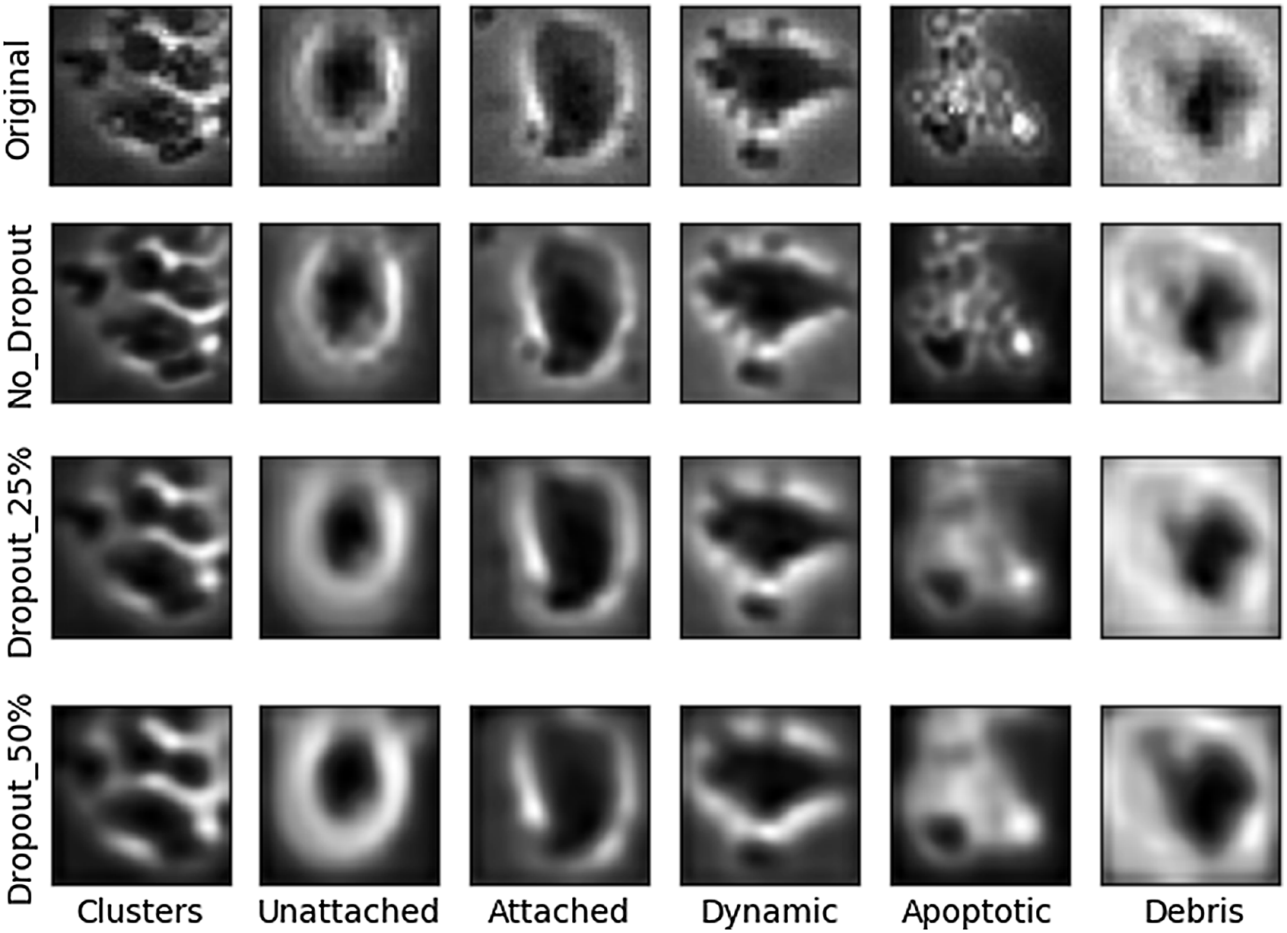
Visual comparison of images reconstructed using our approach of ensemble training versus 25% and 50% dropout rate.

Using an ensemble of classifiers is similar to using dropout during training, but they are not the same.^35^ Ensemble training focuses on training each network with a different subset of data while dropout reduces feature spaces randomly. Although both ensemble method and dropout can generalize the network, the former influences the model with data and the latter manipulates the extracted features. The proposed method uses a simple subnetwork, and each subnetwork was trained independently; therefore, dropout was not considered in each subnetwork. Most importantly, data-driven model preserves all essential features for reconstructing the input image in a simple autoencoder network. Figure 10 shows the comparison of the reconstructed images with and without dropout. It can be seen that when we use dropout the reconstructed images are blurrier due to missing feature information.

### 3.2 Performance Measures

For performance analysis and comparison, we used the confusion matrix for evaluation.^36^ The following equations show the calculations for the overall and individual classification accuracy from the confusion matrix. The average classification rate and individual true positive rate (TPR) are given by the following equations:

$$ACR=\frac{1}{N}\overset{N_{\text{class}}}{\underset{i=1}{\sum }}CM_{ii},$$ (3)

$$TPR_{j}=\frac{1}{N_{j}}\overset{N_{\text{class}}}{\underset{i=1}{\sum }}CM_{ij}.$$ (4)

It is worth noting that $CM_{ii}$ is an ii’th element in the confusion matrix CM. CM is an element of $R^{N_{\text{class}}\times N_{\text{class}}}$ where $N_{\text{class}}$ is the total number of classes. N is the total number of evaluated observations. $TPR_{j}$ is the true positive rate/recall for the j’th class. $N_{j}$ is the total number of samples in the j’th class. $CM_{ij}$ is the element of CM in the i’th row and j’th column.

There are three different categories of accuracies in evaluating the performance of a model: (1) training accuracies, (2) validation accuracies, and (3) out-of-sample testing accuracy. Training and validation accuracies refer to cross validation accuracies for training and validating sets, respectively. The out-of-sample testing accuracy is slightly different than the validation scheme. Once the best model parameters are learned from the model selection process, the final model is obtained with the entire training dataset and the best parameters. This final model is then used to evaluate the performances of the testing dataset, and it produces the out-of-sample accuracy. Typically, training and validation accuracies show us the estimated bias and variance in the final model while out-of-sample testing accuracy shows the true variance in the final model.

### 3.3 Experimental Results

The proposed RandNet is compared with the state-of-the-art methods as reported in Table 2. The top two performers are the proposed RandNet and the fused CNN triplet.^19^ The proposed RandNet has 97.23% mean accuracy in a five-fold cross validation and a seemingly low standard deviation in its validation results. The reason that both RandNet and fused CNN triplet outperformed other methods is that additional data are being used. Both aforementioned methods were trained with data other than the given labeled data. The RandNet used unlabeled data to pre-train its subnetworks and then fine-tuned it with the labeled data. On the other hand, fused CNN triplet^19^ used both synthetic data and real labeled data in training. ResNets,^37^ VGGs,^29^ and AlexNet^30^ were trained with only labeled data. Consequently, they seem to have higher variance in their performances. They also perform worst in out-of-sample testing, as shown in Table 3.

**Table 2:** Five-fold cross-validation results.

Method	Mean accuracy %	STD %
Fused CNN triplet^19^	95.14	0.91
ResNet18^37^	92.16	2.25
ResNet34^37^	92.52	3.34
ResNet50^37^	89.38	2.26
VGG-16^29^	91.78	3.35
VGG-19^29^	93.60	2.48
AlexNet^30^	93.23	2.98
**RandNet**	**97.23**	**0.94**

**Table 3:** Testing data results.

Method	Accuracy %
Fused CNN triplet^19^	95.83
ResNet18^37^	87.59
ResNet34^37^	88.20
ResNet50^37^	86.17
VGG-16^29^	88.29
VGG-19^29^	89.46
AlexNet^30^	87.41
**RandNet**	**96.28**

## 4 Discussions

When comparing with ResNets, VGGs and AlexNet, the proposed method outperformed these methods by at least 6% as shown in Table 3. The performance of these other methods was close within their individual standard deviations. The proposed method has a significantly lower standard deviation than ResNets, VGGs and AlexNet. Therefore, the proposed method still performed better in out-of-sample testing. Since the proposed method incorporated the concept of bagging and used 33 random subnetworks, the proposed method has a low standard deviation.

When comparing with fused CNN triplet,^19^ RandNet outperformed fused CNN triplet in both five-fold cross validation and out-of-sample testing. As shown in Table 2, RandNet was about 2% better than fused CNN triplet in validation results. In terms of out-of-sample testing, the proposed method had a slight 0.45% lead on fused CNN triplet as shown in Table 3. The confusion matrix of the proposed method on the testing dataset is shown in Table 4. The proposed method also outperformed fused CNN triplet of Ref. 19 in terms of training cost. RandNet’s computational cost in training is significantly lower than that of fused CNN triplet. According to Theagarajan et al.,^18^ fused CNN triplet used an additional 240,000 synthetic images for training, 40,000 for each class. Fused CNN triplet took about a month for synthetic image generation and about four days for final model building. On the other hand, the proposed RandNet had about 5 h of training time, and used only 27,603 unlabeled images for pre-training the encoder network. The proposed method was implemented on a desktop with 3.4 GHz Intel(R) Core i7-3770 CPU and NVIDIA GeForce GTX 1070 GPU.

**Table 4:** Confusion matrix for testing data using RandNet.

Prediction →	Cell cluster	Debris	Unattached cell	Attached cell	Dynamically blebbing cell	Apoptically blebbing cell
Cell cluster	**154**	0	0	2	3	1
Debris	0	**187**	1	0	0	0
Unattached cell	0	0	**121**	0	0	1
Attached cell	0	0	6	**173**	2	0
Dynamically blebbing cell	1	5	0	1	**97**	3
Apoptically blebbing cell	4	1	1	0	1	**123**

### 4.1 Misclassification Samples

The proposed method had at least 93% TPR/recall for each individual class, as shown in Table 5. It performed better in identifying attached cells, with a total of 98.30% recall. However, it performed worst for unattached cells. Unattached cells are generally easy to identify as shown in Fig. 2(c).

**Table 5:** Individual recall for RandNet.

Cell type	Recall %
Cell cluster	96.86
Debris	96.89
Unattached cell	93.80
Attached cell	98.30
Dynamically blebbing cell	94.17
Apoptically blebbing cell	96.09

**Fig. 11:**
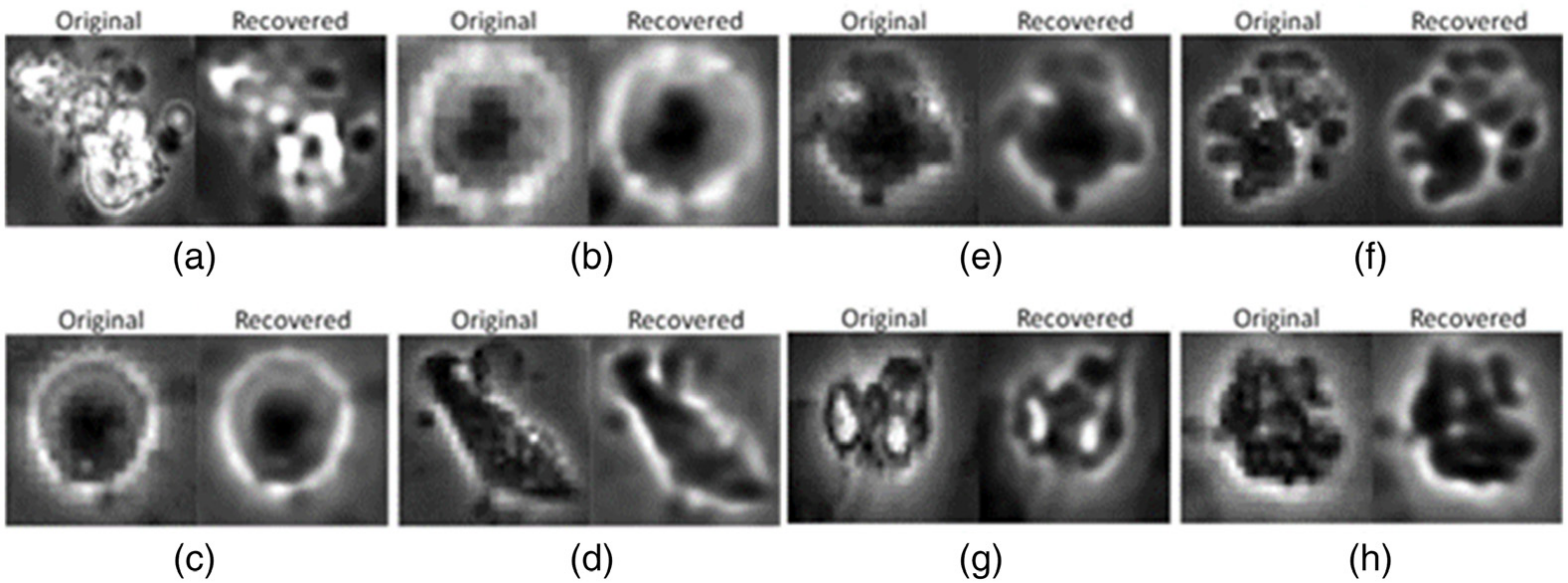
Typical misclassified images in out-of-sample testing: (a) cluster predicted as apoptotic cell; (b) unattached cell predicted as attached cell; (c) unattached cell predicted as attached cell; (d) attached cell predicted as cluster; (e) dynamic blebbing cell predicted as attached cell; (f) dynamic blebbing cell predicted as cluster; (g) debris predicted as apoptotic cell; (h) debris predicted as dynamic blebbing cell. (Note: Recovered images are obtained from the autoencoder network.)

From the typical misclassified images in out-of-sample testing as shown in Fig. 11, we conclude that the blurring effects in the autoencoder network might be the cause for misclassifications. As shown in Figs. 11(b) and 11(c), two unattached cells were blurred out after passing through the autoencoder network. Therefore, these cells looked similar to the attached cells visually. Moreover, this blurring effect might be more significant on the hidden representation generated by the encoder that was used to build the subnetworks.

### 4.2 Additional Experiments

We compared our approach with Mask RCNN;^38^ our approach achieved a Dice coefficient of 0.86, while Mask RCNN achieved 0.92. To train the Mask RCNN, we used 50% of the data for training. A significant difference between the two approaches is that our approach has only four learnable parameters as described in Ref. 3, while Mask RCNN has 43.9 million learnable parameters. Moreover, the approach proposed by Guan et al. can run on a single Intel i7 CPU^3^ while a Nvidia 1080Ti GPU is required to train the Mask RCNN model. Additionally, our detection algorithm is completely unsupervised, whereas Mask RCNN is supervised and requires annotated training data.

Further, we replaced the segmentation component proposed by Guan et al.^3^ in our approach with Mask RCNN^38^ and passed the segmented images as input to our classification component. The classification results and recall for each cell types are shown in Tables 6 and 7, respectively.

**Table 6:** Confusion matrix for RandNet using Mask RCNN as the segmentation component.

Prediction →	Cell cluster	Debris	Unattached cell	Attached cell	Dynamically blebbing cell	Apoptically blebbing cell
Cell cluster	**85**	1	1	0	0	7
Debris	0	**70**	0	2	0	0
Unattached cell	1	0	**62**	0	0	0
Attached cell	8	0	0	**79**	0	0
Dynamically Blebbing cell	0	4	0	0	**37**	0
Apoptically Blebbing cell	0	0	0	0	2	**60**

**Table 7:** Recall of each cell type for RandNet using Mask RCNN as the segmentation component.

Cell type	Recall %
Cell cluster	90.43
Debris	93.33
Unattached cell	98.41
Attached cell	97.53
Dynamically blebbing cell	94.87
Apoptically blebbing cell	89.55

As shown in Table 7, the recall for each cell type was above 89%, and the proposed classification component had an accuracy of 93.79% on the Mask RCNN segmented images. Since the proposed classification component was not trained with samples from Mask RCNN, a small accuracy degradation was expected. The proposed classification component still showed good performance reliability on data samples that were not generated by the proposed segmentation method.

## 5 Conclusions

Automated classification of hESCs in phase contrast videos is essential for a fast quantifiable analysis of hESC behaviors. The proposed RandNet utilized unlabeled data for pre-training, and it incorporated both transfer and ensemble learning concepts. RandNet not only has lower training cost with pre-trained models, but it also can improve performance through fine-tuning with labeled data. It had low performance variance in the cross validation results. This paper has demonstrated that RandNet is an efficient and effective method. In term of efficiency, it uses the combination of subsampling and pre-trained models to generate subnetworks. In term of effectiveness, it is a robust method that provides a generalized solution for hESC classification. Our objective in this paper has been to show that we can use both labeled and unlabeled datasets. This software enables quantitative analysis of changes in and behavior of hESCs in video. In the future, we will explore additional deep networks for building subnetworks. Since the blurring effects of the current simple network affected classification performance, we will explore deeper networks to learn a finer hidden representation for hESC classification.
